# Functional Characterization of Atrophy Patterns Related to Cognitive Impairment

**DOI:** 10.3389/fneur.2020.00018

**Published:** 2020-01-24

**Authors:** Gereon J. Schnellbächer, Felix Hoffstaedter, Simon B. Eickhoff, Svenja Caspers, Thomas Nickl-Jockschat, Peter T. Fox, Angela R. Laird, Jörg B. Schulz, Kathrin Reetz, Imis Dogan

**Affiliations:** ^1^Department of Neurology, RWTH Aachen University, Aachen, Germany; ^2^Research Centre Jülich, Institute of Neuroscience and Medicine (INM-1, INM-7, INM-11), Jülich, Germany; ^3^Institute of Systems Neuroscience, Medical Faculty, Heinrich Heine University, Düsseldorf, Germany; ^4^Institute for Anatomy I, Medical Faculty, Heinrich Heine University, Düsseldorf, Germany; ^5^JARA-BRAIN, Jülich-Aachen Research Alliance, Jülich, Germany; ^6^Iowa Neuroscience Institute, Carver College of Medicine, University of Iowa, Iowa City, IA, United States; ^7^Department of Psychiatry, Carver College of Medicine, University of Iowa, Iowa City, IA, United States; ^8^Research Imaging Center, University of Texas Health Science Center, San Antonio, TX, United States; ^9^Research Service, South Texas Veterans Administration Medical Center, San Antonio, TX, United States; ^10^Department of Physics, Florida International University, Miami, FL, United States

**Keywords:** temporal lobe, parietal lobe, meta-analytical connectivity modeling, resting-state functional connectivity, aging, cognition, neurodegeneration

## Abstract

**Introduction:** Mild cognitive impairment (MCI) is a heterogenous syndrome considered as a risk factor for developing dementia. Previous work examining morphological brain changes in MCI has identified a temporo-parietal atrophy pattern that suggests a common neuroanatomical denominator of cognitive impairment. Using functional connectivity analyses of structurally affected regions in MCI, we aimed to investigate and characterize functional networks formed by these regions that appear to be particularly vulnerable to disease-related disruptions.

**Methods:** Areas of convergent atrophy in MCI were derived from a quantitative meta-analysis and encompassed left and right medial temporal (i.e., hippocampus, amygdala), as well as parietal regions (precuneus), which were defined as seed regions for connectivity analyses. Both task-based meta-analytical connectivity modeling (MACM) based on the BrainMap database and task-free resting-state functional MRI in a large cohort of older adults from the 1000BRAINS study were applied. We additionally assessed behavioral characteristics associated with the seed regions using BrainMap meta-data and investigated correlations of resting-state connectivity with age.

**Results:** The left temporal seed showed stronger associations with a fronto-temporal network, whereas the right temporal atrophy cluster was more linked to cortico-striatal regions. In accordance with this, behavioral analysis indicated an emphasis of the left temporal seed on language generation, and the right temporal seed was associated with the domains of emotion and attention. Task-independent co-activation was more pronounced in the parietal seed, which demonstrated stronger connectivity with a frontoparietal network and associations with introspection and social cognition. Correlation analysis revealed both decreasing and increasing functional connectivity with higher age that may add to pathological processes but also indicates compensatory mechanisms of functional reorganization with increasing age.

**Conclusion:** Our findings provide an important pathophysiological link between morphological changes and the clinical relevance of major structural damage in MCI. Multimodal analysis of functional networks related to areas of MCI-typical atrophy may help to explain cognitive decline and behavioral alterations not tractable by a mere anatomical interpretation and therefore contribute to prognostic evaluations.

## Introduction

Mild cognitive impairment (MCI) is a syndrome marked by a cognitive deficit greater than expected considering age and education level and without relevant impact on daily activities (1–3). MCI is a heterogeneous condition with varying operational definitions, presumably originating from different etiologies and, importantly, may be the precursor of emerging dementia with an annual conversion rate of up to 10% (4, 5). Hence, improving our understanding of early processes of cognitive decline, behavioral symptoms, and degenerative alterations is highly relevant, particularly given that dementia is often caused by irreversible cell degeneration.

Structural brain changes may be observed at an early stage of cognitive decline (6, 7). In order to identify the common neuroanatomical substrates of MCI as a widely defined syndrome, Nickl-Jockschat et al. (8) performed a quantitative meta-analysis of voxel-based morphometry (VBM) studies comparing MCI patients diagnosed via the Petersen criteria (1, 9) with healthy controls. Consistent structural changes across studies were found in three clusters mainly encompassing bilaterally the hippocampus and amygdala, and the parietal precuneus (8). Gray matter reductions in the amygdala, hippocampus and thalamus were additionally associated with decreased cognitive performance (8). Although different pathologies may underlie MCI, this convergent temporo-parietal atrophy pattern can be considered to reflect a common neuropathological denominator of cognitive impairment (8). However, a comprehensive understanding of the clinical profile linked to such alterations should consider the complex interactions within neuronal circuits formed by or emanating from areas susceptible to disease pathology. This is particularly true given the notion that neurogenerative disorders represent diseases with distinct patterns of network disintegration (10, 11). Moreover, neurodegenerative diseases have been described as “nexopathies” (Latin nectere, tie) referring to the spread of pathogenic protein abnormalities via large-scale brain networks and differential intrinsic network vulnerability (12). In this context, the regions of convergent volume loss in MCI identified by Nickl-Jockschat et al. (8), which are also parts of the default mode network (DMN), can be considered as network nodes particularly vulnerable in MCI. Computational models have emphasized the role of structural network hubs as highly interconnected neural regions that are important for the integration and segregation of brain networks (13, 14). A disruption of such circuits due to morphological changes will be detrimental to network functionality, which in turn may likely lead to clinical manifestations going beyond a merely anatomical interpretation of circumscribed atrophic regions.

In the current study we aimed to functionally and behaviorally characterize the atrophy pattern previously observed in MCI and delineate ensuing functional networks connected to these regions that are prone to disruption in MCI. To achieve this, regions of convergent volume loss as identified by Nickl-Jockschat et al. (8) were defined as seed regions and subjected to functional connectivity modeling using different modalities. (i) First, functional connectivity was assessed using task-based meta-analytical connectivity modeling (MACM), which identifies stimuli-driven networks during task performance using an extensive amount of meta-data of functional imaging studies stored in the BrainMap database (15). (ii) Second, we employed task-free resting-state functional MRI (fMRI) data of a large sample of older healthy probands derived from the 1000BRAINS study (16) to assess endogenously controlled functional connectivity profiles coupled with respective atrophy seeds. This non-clinical cohort enabled the identification of characteristic networks that are expected to co-activate with our seed regions in an aging population and may be disrupted when morphological changes occur. Additionally, the combination of both approaches allowed the analysis of convergence between both task-driven and task-independent functional networks related to the regions of atrophy, representing a more robust estimation of “core” connectivity profiles across different modalities (17). (iii) In a further step, again using meta-data from BrainMap we aimed to behaviorally characterize the atrophy nodes by inferring from the specific behavioral domains and paradigms that consistently elicited activation in these regions in previously published functional imaging studies. (iv) Finally, as MCI is an age-associated disease and there are connectivity alterations with increasing age (18), we performed correlation analyses between age and resting-state connectivity of MCI-typical atrophy regions. This allows a better differentiation between age-related connectivity changes and those expected to be associated with MCI.

Given the several definitions of the MCI syndrome over the last decades, we note that in the current study we focused on the definition by Petersen (9) that any cognitive domain may be affected. While different causes other than neurodegenerative processes (e.g., vascular diseases, depression) may lead to MCI, use of this broader definition enables the characterization of an early temporo-parietal atrophy pattern representing a common neuropathological substrate of cognitive impairment (8).

## Materials and Methods

### Seed Regions: Regions of Convergent Atrophy in MCI

**Figure 1:**
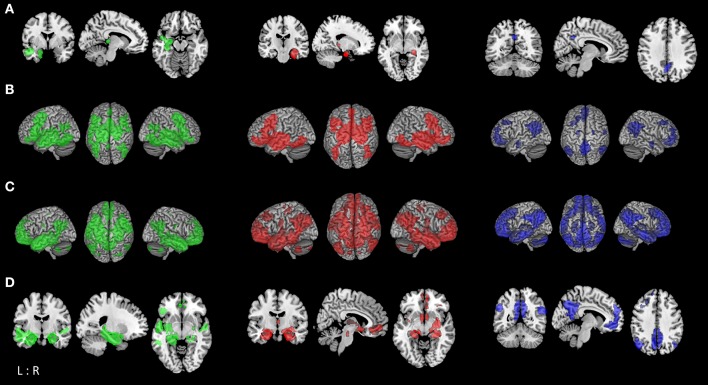
Functional connectivity modeling of MCI-atrophy seeds. **(A)** Location of the seed regions (*left (green)*: left temporal seed; *middle (red)*: right temporal seed; *right (blue)*: parietal seed [same color coding for **(B–D)**]) showing convergent evidence of atrophy as revealed by coordinate-based meta-analysis across voxel-based morphometry studies in MCI (8). **(B)** Task-based brain-wide co-activation maps of the respective seed regions as revealed by meta-analytic connectivity modeling (MACM; cluster-level FWE corrected at *p* < 0.05, *p* < 0.001 at voxel-level). **(C)** Resting-state connectivity of respective seeds (FWE corrected at *p* < 0.05). **(D)** Conjunction between MACM and resting-state connectivity of respective seed regions using minimum statistic (19).

Functional connectivity analysis was based on seed regions identified by Nickl-Jockschat et al. (8), representing areas of common consistent atrophy in MCI (Figure 1A). In this previously published coordinate-based meta-analysis, 22 VBM studies comparing in total 917 MCI patients (predominantly amnestic MCI) with 809 healthy controls were included and three supra-threshold clusters of convergent atrophy in MCI were identified: The largest cluster (cluster extent k_E_: 2407 voxels, MNI-coordinates of cluster maxima in x/y/z: −22/−8/−22) was localized in the left medial temporal lobe, including the hippocampus (cornu ammonis) and laterobasal amygdala. The second cluster (k_E_: 1984 voxels, 24/−8/−20) was located on the right temporal lobe encompassing the laterobasal amygdala, fascia dentata of the hippocampus and parahippocampal gyrus. The third cluster (k_E_: 269 voxels, 2/-54/32) was mainly located in the precuneus extending to the posterior cingulate cortex [PCC; (8)].

### Task-Based Meta-Analytic Connectivity Modeling (MACM)

Task-based functional connectivity of MCI-typical atrophy seeds was calculated via meta-analytic connectivity modeling [MACM; (20–22)] based on the BrainMap database [www.brainmap.org; (15, 23, 24)]. By examining the functional connectivity profile of co-activations reported across the entire brain, we aimed to identify functional networks connected with these atrophy regions and therefore most likely to be disrupted in patients with MCI.

MACM assesses the brain-wide co-activation pattern of an anatomical region across a large number of functional neuroimaging results in healthy individuals stored in BrainMap (please see Section Behavioral Characterization for more information on paradigm classes and behavioral domains) and identifies significant areas of above-chance co-activation with this seed region. For this, all eligible experiments reporting at least one activation focus of within-subject effects between conditions were identified. Using the activation likelihood estimation (ALE) approach, convergence across these brain-wide foci was tested for identifying consistent co-activation (i.e., task-based functional connectivity) with the respective seed (15, 25–27). The reported foci were treated as centers of 3D Gaussian probability distributions reflecting the spatial uncertainty associated with each reported set of coordinates (25). The probabilities of all foci reported in the experiments were then combined for every voxel and a modeled activation (MA) map was drawn (27). The union of these maps yielded voxel-wise ALE scores describing the level of convergence at each location in the brain, which were compared to a null-distribution (26). Non-parametric *p*-values for each meta-analysis were thresholded at a cluster-level corrected family-wise error (cFWE) of *p* < 0.05 (uncorrected at the voxel-level with *p* < 0.001). Additionally, we performed contrast analyses of network connectivity between seed regions to assess divergent connectivity profiles between the three atrophy seeds (28). Thereby we aimed to delineate networks showing stronger functional connectivity with one seed region in particular compared to the remaining ones. Contrast analysis results were thresholded at a posterior probability of *p* > 0.95 (k_E_≥50 voxels) for a true difference between the two samples (17). All results were anatomically labeled by reference to probabilistic cytoarchitectonic maps of the human brain implemented in the SPM Anatomy Toolbox (29).

### Task-Independent “Resting-State” Connectivity Modeling

We further performed seed-voxel-wise connectivity analysis of each atrophy seed using resting-state functional MRI (rs-fMRI) data from the 1000BRAINS study (16). The 1000BRAINS study is a longitudinal large-scale imaging study that investigates functional and structural variability of the aging brain, providing a unique source of imaging data in a large sample of healthy older adults. Hence, in order to delineate functional connectivity profiles across different modalities, we examined endogenously controlled resting-state networks co-activating with our seed regions in addition to rather externally-driven task-based connectivity (17). The large 1000BRAINS dataset of older healthy adults further gave us the opportunity to investigate the effect of aging on the functional connectivity of those areas demonstrating morphological changes in MCI.

For the current analysis, we used data from 637 healthy older subjects (mean age 66.7 ± 6.3 SD years, range: 55–85 years; 50.2% male; formal school years 9.9 ± 2.1; vocational/higher education 3.9 ± 2.7 years) with no history of neurological or psychiatric disorders. Only subjects with a score of at least 13 points (mean 15.5 ± 1.9 SD; range: 13–18) in the cognitive screening tool DemTect (30) indicating no signs of early dementia were included. Resting-state fMRI scans of 11:30 min duration were performed while eyes were closed, light switched off, and with the instruction to let the mind wander without thinking of anything in particular and not to fall asleep. Gradient-echo echoplanar images (EPI) were acquired on a 3T Siemens Tim Trio MR scanner (Erlangen, Germany) with the following sequence parameters: TR = 2.2 s, TE = 30 ms, FoV = 200 × 200 mm^2^, flip angle = 90°, voxel resolution = 3.1 × 3.1 × 3.1 mm^3^, 36 slices. Physiological and movement artifacts were removed from the data using FIX [FMRIB's ICA-based Xnoiseifier, implemented in FSL; (31)]. FIX decomposes the data into independent components using FSL melodic and classifies noise components using distinct spatial and temporal features, which are then regressed out of the raw fMRI data. Further processing was performed using SPM12 (http://www.fil.ion.ucl.ac.uk/spm/) and in-house Matlab tools. Images were normalized to the MNI template (32) and smoothed with a 5 mm FWHM Gaussian kernel. In order to reduce spurious correlations, variance that could be explained by nuisance variables was removed, i.e., the six motion parameters derived from image realignment, the first derivate from the realignment parameters, and the mean tissue class signals (gray matter, white matter, CSF) per time-point obtained by averaging across voxels (33, 34). Finally, using a bandpass filter we examined frequencies between 0.01 and 0.08 Hz as meaningful resting-state signal will predominantly be found in these frequencies (35).

Statistical analysis was performed in correspondence to the MACM analysis as described above. Pearson correlation coefficients were transformed into Fisher's Z-scores in a connectivity matrix and tested for consistency across subjects in a second-level ANOVA with age included as a nuisance regressor. We first assessed resting-state connectivity of each atrophy seed separately (FWE corrected at voxel-level with *p* < 0.05). Subsequently, we performed contrast analyses between connectivity networks of the MCI-related atrophy seeds, and additionally calculated correlations between voxel-wise co-activation of each seed region and age (cFWE corrected *p* < 0.05, *p* < 0.001 at voxel-level).

### Conjunction of Task-Based MACM and Task-Free Resting-State Functional Connectivity

We performed conjunction analyses between resting-state and task-based (MACM) functional connectivity maps of seed regions using the minimum statistics (19). The aim here was to identify the brain-wide co-activation profile of each atrophy area in both task-related and task-free states yielding a more robust and mode-independent delineation of networks functionally connected to the seed regions (17, 36).

### Behavioral Characterization

For further differentiation of the seed regions affected in MCI a behavioral characterization was performed. MCI-related atrophy clusters were submitted to functional profiling using meta-data of the BrainMap database. In the BrainMap taxonomy, behavioral domains (BD) describe the specific mental process isolated by the statistical contrast of each archived neuroimaging experiment (23) and include the main categories of cognition, action, perception, emotion, interoception, as well as their related subcategories. Additionally, paradigm classes (PC) define the specific tasks employed in the experiment (for a complete list of taxonomy cf. http://brainmap.org/scribe/). Each cluster was analyzed regarding its associated behavioral domain and paradigm class by computing conditional probabilities (forward [P(activation | domain or paradigm)] and reverse inference [P(domain or paradigm | activation)]) (17, 36). For forward inference, significant over-representation of BD and PC in the experiments activating the respective seed region relative to the overall chance of finding activation in that particular seed across the BrainMap database was assessed using a binomial test at *p* < 0.05, FDR corrected (20, 28). For the reverse inference, a seed's functional profile was determined by identifying the most probable BD and PC given activation in a particular cluster using chi-square tests (*p* < 0.05, FDR corrected) (20). Statistical overrepresentation of a specific behavioral domain and paradigm class allowed the identification of the functional role of the selected seed region (36).

## Results

### Task-Based Functional Connectivity of Seed Regions (MACM)

The co-activation patterns revealed by MACM were similar for the left and right temporal seed, and demonstrated convergent connectivity with the hippocampus, amygdala, thalamus, and striatum (caudate nucleus, putamen), and cortically in the posterior medial frontal gyrus (supplementary motor area, SMA), inferior frontal gyrus, middle orbital and rectal gyrus, insula, fusiform gyrus (FG4, FG2), right inferior temporal and inferior occipital gyrus, as well as cerebellum (lobule VI). The left temporal seed additionally demonstrated co-activation with bilateral middle and inferior temporal areas, left cerebellum (lobule VI), left inferior parietal lobe (IPL, mainly PGa, PFm), parietal operculum and precuneus. MACM of the parietal cluster showed convergent co-activation with the precuneus, posterior cingulate cortex (PCC), middle orbital and rectal gyrus, superior medial and anterior cingulate cortex (ACC), IPL (PGa, PFm, PGp), angular gyrus, left middle and superior frontal gyrus, right middle temporal gyrus as well as amygdala and hippocampus (Figure 1B; Table S-1).

**Figure 2:**
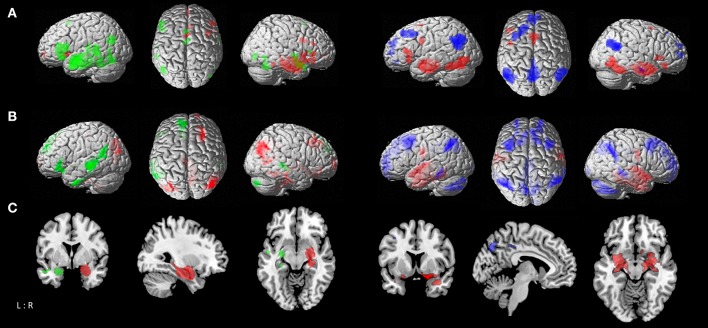
Comparison of functional connectivity maps between atrophy seeds. **(A)** *left*: MACM contrasts between left and right temporal seeds, with *green* areas showing stronger connectivity to left temporal seed, and *red* areas showing stronger connectivity to right temporal seed; *right*: MACM contrasts of parietal seed against the conjunction of left and right temporal seeds, with *blue* areas showing stronger connectivity to parietal seed, and *red* areas showing stronger connectivity to both right and left temporal seeds. **(B)** *left*: Resting-state connectivity contrasts between left and right temporal seeds; *right*: contrast of parietal seed against the conjunction of left and right temporal seeds [color coding as in A]. **(C)** Conjunction of MACM and resting-state contrast maps; *left*: contrasts between left and right temporal seeds; *right*: contrast of parietal seed against the conjunction of left and right temporal seeds [color coding as in **(A)**].

In the statistical comparison of both temporal seeds the left temporal cluster showed in contrast to the contralateral one stronger connectivity with the bilateral fusiform gyrus (FG3, FG4), middle temporal gyrus and cerebellum (lobules VI-VII), left inferior temporal and frontal gyrus, inferior occipital and angular gyrus. The right temporal seed had a stronger focus on the bilateral basal ganglia (caudate nucleus, putamen, pallidum), middle orbital gyrus and right middle cingulate cortex. Contrasting both temporal seeds with the parietal one delineated co-activation of temporal seeds in the bilateral hippocampus, amygdala, striatum, fusiform gyrus, inferior occipital gyrus, middle cingulate cortex, cerebellum (lobule VI) and the left inferior frontal gyrus. The parietal seed revealed stronger convergence compared to the temporal seeds in the middle orbital and rectal gyrus areas, superior medial frontal and ACC, angular gyrus, and left middle and superior frontal gyrus (Figure 2A; Table S-2).

### Task-Free Functional Connectivity of Seed Regions (Resting-State fMRI)

Resting-state connectivity modeling results of the left and right temporal seeds were again very similar, both showing co-activation with the hippocampus, amygdala, thalamus, precuneus, PCC, angular and middle temporal gyrus, fusiform (FG3, FG4) and rectal gyrus. On the other hand, the right temporal seed co-activated with the superior frontal gyrus, pre- and post-central gyrus, right superior occipital and angular gyrus. While both seeds showed connectivity with cerebellar lobules IX-X and VIIa, the left temporal seed additionally co-activated with lobules IV–VI. The parietal seed exhibited a more widespread connectivity pattern than in the MACM analysis. This included orbital gyri, cingulate cortex, medial frontal and fronto-insular cortex, angular gyrus, middle and inferior temporal gyrus, hippocampus and amygdala, and the cerebellar lobule IX (Figure 1C; Table S-3).

Contrasting the left temporal seed with the contralateral one delineated stronger co-activation in the middle temporal gyrus, left inferior and superior frontal gyrus, insula, angular gyrus, inferior temporal gyrus, putamen, and right cerebellum (lobule VIIa), while the right temporal seed delineated more right-hemispheric convergence in the temporal and fusiform gyrus (FG4, FG3), angular gyrus, precuneus and middle cingulate cortex, middle and superior frontal gyrus, middle orbital gyrus, and cerebellum (lobule IX, X), as well as the bilateral striatum (caudate nucleus and putamen). The temporal seeds had a stronger focus in comparison to the parietal one on the post-central gyrus, fusiform gyrus (FG3, FG4), superior temporal gyrus, and left inferior temporal gyrus in addition to hippocampal and amygdalae regions. The parietal seed on the other hand had more pronounced connectivity with the precuneus, ACC and PCC, superior and middle frontal gyrus, angular gyrus, middle temporal gyrus, thalamus and cerebellar lobules (Figure 2B; Table S-4).

### Conjunction of Task-Based and Task-Free Functional Connectivity

To outline a more robust and mode-independent co-activation profile of each atrophy area we performed conjunction analyses between resting-state and MACM maps of seed regions. Here we found convergent co-activation with the left temporal cluster in the left inferior frontal gyrus, insula, PCC and precuneus, bilateral middle, and superior temporal gyrus in addition to the hippocampus, amygdala, and thalamus. The fusiform gyrus (FG3, FG4), rectal gyrus and left parahippocampal gyrus were co-activated with both temporal clusters, while the right inferior frontal and occipital gyrus, putamen, and pallidum demonstrated connectivity only with the ipsilateral temporal seed. The parietal cluster demonstrated convergent connectivity with the precuneus, PCC and ACC, angular gyrus, rectal/middle orbital gyrus, left middle and superior frontal gyrus, and right middle temporal gyrus (Figure 1D; Table 1). Conjunction analysis of all three seed regions across modalities revealed common co-activation within the bilateral rectal gyrus and hippocampus.

**Table 1:** Functional connectivity of MCI-atrophy seeds.

**Cluster #**	**k_**E**_**	MNI co-ordinates^*^			**Lat**.	**Macroanatomical and cytoarchitectonic region**
		**x**	**y**	**z**		
**Temporal left**						
Cluster 1	5375	−24	−12	−34	L	Hippocampus (CA, EC, SUB, DG), parahippocampal gyrus, amygdala (LB, SF, CM), thalamus, superior and middle temporal gyrus, fusiform gyrus, insula lobe
Cluster 2	1962	42	14	−34	R	Hippocampus (CA, EC, SUB, DG), amygdala (LB, SF, CM), thalamus, superior and middle temporal gyrus
Cluster 3	399	−42	26	−16	L	Inferior frontal gyrus (p. opercularis, p. orbitalis)
Cluster 4	292	−8	−56	6	L	Precuneus, PCC, calcarine gyrus
Cluster 5	255	−48	−68	16	L	Middle temporal gyrus
Cluster 6	116	−2	40	−20	L	Rectal gyrus
Cluster 7	79	40	−44	−28	R	Fusiform gyrus
Cluster 8	66	4	42	−20	R	Rectal gyrus
**Temporal right**						
Cluster 1	3307	30	−10	−31	R	Hippocampus (CA, SUB, DG, EC, HATA), amygdala (LB, SF, CM), thalamus, fusiform gyrus, pallidum, putamen
Cluster 2	2379	−24	−12	−32	L	Hippocampus (CA, SUB, DG, EC, HATA), amygdala (LB, SF, CM), thalamus, fusiform gyrus, parahippocampal gyrus
Cluster 3	300	42	−48	−28	R	Fusiform gyrus
Cluster 4	240	2	42	−22	R	Rectal gyrus
Cluster 5	160	−2	42	−22	L	Rectal gyrus
Cluster 6	114	42	26	18	R	Inferior frontal gyrus (p. orbitalis)
Cluster 7	91	6	−2	0	R/L	Medial thalamus
Cluster 8	52	46	−74	−2	R	Inferior occipital gyrus
**Precuneus**						
Cluster 1	1442	−10	−56	2	L	Precuneus, PCC, MCC, lingual gyrus
Cluster 2	1221	−52	−68	14	L	Angular gyrus. IPL
Cluster 3	1052	10	−56	10	R	Precuneus, PCC, MCC, calcarine gyrus
Cluster 4	962	−2	46	−22	L	Rectal gyrus, middle orbital gyrus, ACC
Cluster 5	805	50	−70	16	R	Angular gyrus, IPL
Cluster 6	527	−36	18	40	L	Superior and middle frontal gyrus
Cluster 7	354	2	42	−20	R	Rectal gyrus
Cluster 8	168	56	−8	−28	R	Middle temporal gyrus

*Conjunction of both task-based (MACM) and task-free (resting-state) functional connectivity maps of each MCI-atrophy seed (cluster-level FWE corrected at p < 0.05; cluster-forming threshold p < 0.001)*.

* *Cluster-maxima in MNI space. k_E_, cluster extent; Lat., laterality; L, left; R, right; CA, cornu ammunis; EC, entorhinal cortex; SUB, subiculum; DG, dentate gyrus; HATA, hippocampus–amygdala-transition-area; LB, laterobasal; SF, superficial; CM, centromedial; MCC, middle cingulate cortex; PCC, posterior cingulate cortex*.

Contrasting both temporal seeds, the left temporal cluster revealed stronger co-activation with the left putamen, middle temporal gyrus, as well as inferior frontal gyrus. The right temporal cluster showed more co-activation in the right putamen, thalamus and bilateral caudate nucleus. Both right and left temporal seeds exhibited stronger connectivity compared with the parietal seed in the hippocampus, amygdala, and fusiform gyrus. The parietal seed demonstrated co-activation primarily in cortical structures including the left middle frontal gyrus and middle occipital gyrus, and bilateral angular gyrus (Figure 2C; Table 2).

**Table 2:** Comparison of functional connectivity maps of MCI-atrophy seeds.

**Cluster #**	**k_**E**_**	MNI co-ordinates^*^			**Lat**.	**Macroanatomical and cytoarchitectonic region**
		**x**	**y**	**z**		
**Contrast: Left** **>** **right temporal seed**						
Cluster 1	290	−57	−37	1	L	Middle temporal gyrus
Cluster 2	205	−48	28	−5	L	Inferior frontal gyrus (p. orbitalis)
Cluster 3	17	−30	−6	−6	L	Putamen
**Contrast: Right** **>** **left temporal seed**						
Cluster 1	105	38	−12	−12	R	Thalamus, putamen
Cluster 2	39	21	−27	−3	R	Thalamus
Cluster 3	31	−8	−4	−12	L	Caudate nucleus
Cluster 4	23	10	16	−14	R	Caudate nucleus
**Contrast: [right and left] temporal seeds** **>** **parietal seed**						
Cluster 1	1059	−26	−12	−34	L	Hippocampus (CA, SUB), amygdala (LB, SF, CM), fusiform gyrus
Cluster 2	991	28	−8	−34	R	Hippocampus (CA, SUB), amygdala (LB, SF, CM), fusiform gyrus
Cluster 3	181	−36	−48	−26	L	Fusiform gyrus
Cluster 4	112	40	−44	−28	R	Fusiform gyrus
**Contrast: Parietal seed** **>** **[right and left] temporal seeds**						
Cluster 1	217	−58	−54	30	L	Angular gyrus
Cluster 2	177	−8	−72	32	L	Precuneus
Cluster 3	175	−36	18	40	L	Middle frontal gyrus
Cluster 4	80	60	−50	24	R	Angular gyrus
Cluster 5	23	−6	−34	40	L	MCC

*Conjunction of both task-based (MACM) and task-free (resting-state) functional connectivity maps of each MCI-atrophy seed (cluster-level FWE corrected p < 0.05, p < 0.001 at voxel-level)*.

* *Cluster-maxima in MNI space. k_E_, cluster extent; Lat., laterality; L, left; R, right; CA, cornu ammunis; EC, entorhinal cortex; SUB, subiculum; DG, dentate gyrus; HATA, hippocampus–amygdala-transition-area; LB, laterobasal; SF, superficial; CM, centromedial, MCC, middle cingulate cortex*.

### Behavioral Characterization of Seed-Regions

**Figure 3:**
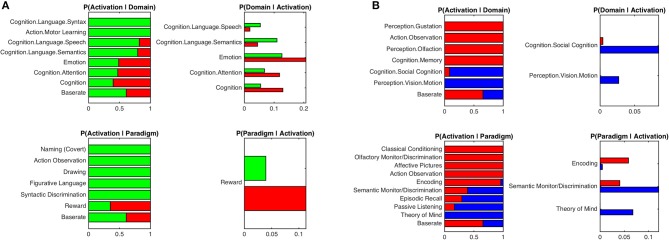
Functional characterization of atrophy seeds by behavioral domains and paradigm classes. **(A)** Functional characterization by behavioral domains and paradigm classes of left temporal seed (*green*) in contrast to right temporal seed (*red*). **(B)** Functional characterization by behavioral domains and paradigm classes of parietal seed (*blue*) in contrast to the conjunction of left and right temporal seeds (*red*). Bar plots show significant associations (at *p* < 0.05, FDR corrected) of behavioral domains and paradigm classes from the BrainMap meta-data given observed brain activity (and vice versa); the x-axis indicates relative probability values.

Behavioral characterization of the MCI-atrophy seeds using meta-data from the BrainMap database indicated that activation in the left temporal cluster in contrast to the right temporal seed was elicited by cognitive domains related to language syntax, speech and semantics as well as motor learning. The right temporal cluster showed high probability of activation in the domains of emotion and attention. Activation was more likely in the left temporal cluster given paradigm classes of naming, action observation, drawing and figurative language as well as syntactic discrimination (Figure 3A), while the right temporal cluster demonstrated a focus on the paradigm class of reward. In order to outline distinctive behavioral associations of temporal vs. parietal atrophy regions, we contrasted both temporal seeds against the parietal seed and found predominance of the domains perception (gustation and olfaction), action (observation), and cognition (memory) for the temporal clusters. Paradigms were classical conditioning, olfactory discrimination, action observation, encoding, and affective pictures. Activation in the parietal cluster was elicited given the domains social cognition and perception of motion, and paradigm classes of semantic discrimination, episodic recall, passive listening, and theory of mind (Figure 3B).

### Age-Dependent Functional Connectivity of Seed-Regions

**Figure 4:**
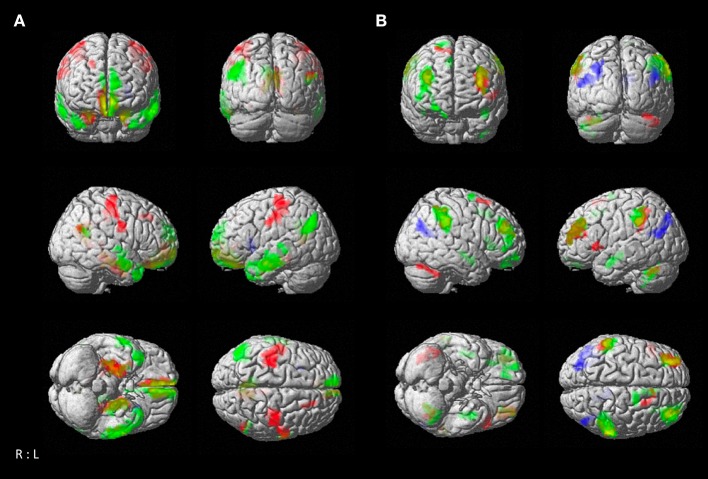
Resting-state connectivity correlation of atrophy seeds with age. **(A)** Negative correlation between age and brain-wide resting-state connectivity of seeds (*green*: left temporal seed; *red*: right temporal seed; *blue*: parietal seed). **(B)** Positive correlation between age and brain-wide resting-state connectivity of seeds with age [color coding as in **(A)**]. Results are cluster-level FWE corrected at *p* < 0.05 (*p* < 0.001 at voxel-level).

Resting-state functional connectivity decreased with higher age between the left temporal cluster and the hippocampus, amygdala, orbitofrontal area, medial frontal cortex, fusiform gyrus, middle and inferior temporal gyrus, angular gyrus, and precuneus. A similar pattern was observed for the right temporal seed, which additionally showed negative associations with age in precentral and post-central gyrus, while the connectivity of the left temporal seed with right temporal areas was negatively correlated with age. The parietal seed only revealed a decrease in connectivity to the anterior insula with higher age (Figure 4A; Table 3). We also found positive correlations between age and resting-state connectivity. Both the left and right temporal seed showed increased connectivity with higher age mainly with the lateral prefrontal cortex, inferior parietal lobe, insula and cerebellum (lobules VI, VIIa). The parietal cluster demonstrated increased co-activation with the middle temporal gyrus, middle occipital gyrus, angular gyrus and the precuneus with higher age (Figure 4B; Table 3).

**Table 3:** Resting-state fMRI connectivity correlations with age.

**k_**E**_**	MNI co-ordinates^*^			**Lat**.	**Anatomical region**	**k_**E**_**	MNI co-ordinates^*^			**Lat**.	**Anatomical region**
	**x**	**y**	**z**				**x**	**y**	**z**		
**Left temporal seed: Negative correlation**						**Positive correlation**					
1385	−4	64	−8	L	Middle orbital, rectal gyrus, superior medial gyrus	1163	60	−36	36	R	Angular gyrus
1108	−60	−6	−22	L	Middle and inferior temporal gyrus	791	36	50	28	R	Middle frontal gyrus, inferior frontal gyrus
845	−26	−14	−22	L	Hippocampus (DG, SUB, CA, HATA), amygdala (CM, SF), parahippocampal gyrus	527	−32	46	32	L	Middle frontal gyrus
835	−50	−70	36	L	Angular gyrus	440	−56	−36	52	L	Inferior parietal lobe, supramarginal gyrus
633	−8	−54	10	L	Precuneus, PCC, calcarine gyrus	397	−28	−62	−30	L	Cerebellum (Crus 1, VI, VIIa)
488	18	−8	−20	R	Hippocampus (HATA, SUB, CA), amygdala (SF, LB), fusiform gyrus	283	−40	0	−20	L	Insula
449	2	38	−22	R	Rectal gyrus, middle orbital gyrus	178	26	60	−16	R	Middle orbital gyrus, superior orbital gyrus
372	6	−52	16	R	Precuneus, calcarine gyrus	173	6	20	46	R	Superior medial gyrus
351	62	0	−20	R	Middle and superior temporal gyrus	138	44	−12	−10	R	Superior temporal gyrus, insula
256	40	12	−36	R	Medial temporal pole	130	34	20	12	R	Insula lobe
140	4	62	10	R	Superior medial gyrus, ACC	115	20	−4	70	R	Superior frontal gyrus
130	48	−54	18	R	Middle temporal gyrus, angular gyrus						
102	−66	−24	2	L	Middle temporal gyrus						
**Right temporal seed: Negative correlation**						**Positive correlation**					
1197	20	−8	−22	R	Hippocampus (CA. SUB, DG), amygdala (LB, CM), fusiform gyrus	706	−32	50	30	L	Middle frontal gyrus, inferior frontal gyrus (p. triangularis)
1119	−36	−28	58	L	Precentral gyrus (Area 4a), post-central gyrus(1,3b)	367	−56	−42	52	L	Angular gyrus
774	6	−54	14	R	Precuneus, calcarine gyrus, lingual gyrus, PCC	282	38	−70	−24	R	Cerebellum (Crus 1, VIIa)
704	4	50	−16	R	Rectal, middle orbital gyrus, superior medial gyrus	273	64	−40	42	R	Supramarginal gyrus, angular gyrus
694	−22	−12	−24	L	Hippocampus (CA, DG, SUB, HATA, EC), amygdala (LB, SF, CM), fusiform gyrus	256	36	38	24	R	Middle frontal gyrus
573	46	−20	58	R	Post-central gyrus (1, 3b), precentral gyrus (4a, p)	197	−34	−56	−30	L	Cerebellum (Crus 1, VI, VIIa)
454	−4	64	−6	L	Middle orbital gyrus, rectal gyrus, ACC	126	−48	20	4	L	Inferior frontal gyrus (p. triangularis)
422	−10	−56	10	L	Precuneus, PCC	108	18	18	64	R	Superior frontal gyrus
258	56	−14	44	R	Post-central gyrus (Area 1, 3b)						
114	50	−60	28	R	Angular gyrus						
107	22	30	42	R	Middle and superior frontal gyrus						
106	−62	−2	−22	L	Middle and inferior temporal gyrus						
**Parietal seed: Negative correlation**						**Positive correlation**					
98	−26	14	6	L	Insula lobe	610	−52	−68	16	L	Middle temporal, middle occipital, angular gyrus
						260	44	−68	28	R	Middle occipital, angular, middle temporal gyrus
						159	16	−44	28	R	Precuneus, PCC

*Correlation between age and resting-state fMRI connectivity of each MCI-atrophy seed (cluster-level FWE corrected at p < 0.05; cluster-forming threshold p < 0.001)*.

* *Cluster-maxima in MNI space. k_E_, cluster extent; Lat., laterality; L, left; R, right; CA, cornu ammunis; EC, entorhinal cortex; SUB, subiculum; DG, dentate gyrus; HATA, hippocampus–amygdala-transition-area; LB, laterobasal; SF, superficial; CM, centromediale; ACC, anterior cingulate cortex; PCC, posterior cingulate cortex; Id, insular dysgranular area; Ig, insular granular area; IPL, inferior parietal lobule*.

## Discussion

Based on convergent morphological changes in MCI, we conducted task-based and task-free connectivity analyses and identified neural networks giving further insight into the pathophysiological relevance of structural damage in MCI. For each atrophy seed, we observed widespread but also distinct connectivity patterns and respective behavioral characteristics. While the left temporal seed showed stronger associations with a fronto-temporal network and an emphasis on language generation, the right temporal atrophy cluster was more linked to cortico-striatal regions and the domains of emotion and attention. The parietal seed demonstrated strong connectivity within the DMN, in particular with frontoparietal regions and was associated with introspection and social cognition. These networks suggest increased vulnerability in MCI due to beginning degenerative processes in important hub centers functionally connected to these areas and may underlie the heterogeneous clinical picture in this syndrome. Correlation analysis revealed both decreasing and increasing functional connectivity of atrophy seeds with higher age that may augment pathological processes but also indicates potential compensatory mechanisms of functional reorganization.

### Functional Connectivity of Temporal Atrophy Seeds

Investigations into cerebral network characteristics are important for our understanding of neurodegenerative diseases, in particular given the notion of disease spreading along neuronal pathways rather than by spatial proximity (37, 38). More important than proximity seems to be the functional association with certain hub regions of the brain (39). To this end we assessed functional connectivity patterns of regions at risk in MCI that may facilitate identification of disease-related network disruptions. Functional connectivity analysis of temporal seeds revealed widespread but also distinct patterns of co-activation related to each temporal seed. In particular the *right* temporal seed showed convergent co-activation with a fronto-striatal network including orbitofrontal regions, the caudate nucleus, putamen and pallidum. This is of clinical importance since the striatum is involved not merely in motor functions but also in executive control and motivational processes, such as experience of reward and punishment (40–42). Convergent functional connectivity of the right temporal seed with the medial orbitofrontal cortex (OFC) additionally suggests a functional link to emotional control and reward processing (43–45). According to the hypothesis of network-spreading in neurodegenerative diseases, functionally connected regions are subject to a higher risk of disease-related vulnerability. Hence, this convergent functional connectivity pattern of morphologically affected right temporal regions may help to explain the high prevalence of psychiatric disorders such as depression in MCI patients (46), since neuronal pathways responsible for the generation of reward experiences and emotional control mechanisms may be disrupted. Behavioral analysis based on BrainMap meta-data emphasized these results showing associations of the right temporal seed with the paradigm class of reward, and the domains of emotion and attention. Nonetheless, the domain of emotion and reward processes are not entirely independent from each other and can be interlinked in the BrainMap taxonomy, whereby a clear distinction between studies eliciting activation in this region is difficult.

The *left* temporal seed on the other hand had a stronger functional connectivity with a fronto-temporal network, and behavioral decoding of the left temporal seed supported an emphasis on speech, semantic and syntax. Semantic deficiencies have been reported in MCI-patients (47) and can be observed in neurodegenerative dementias. In particular, the convergent co-activation with the left inferior frontal gyrus, both in task-dependent and task-independent analysis, suggests susceptibility in pathways playing a role in speech generation, and accords with observed vulnerability to atrophy of the inferior frontal gyrus in patients with Alzheimer's disease (48). Task-dependent analysis additionally revealed functional connectivity between the left temporal seed and cerebellar lobule VI and VII. This is of interest as the cerebellum is involved in a broad range of cognitive domains (49–51). Particularly right lobule VI, which connects to the left cerebral hemisphere, is involved in language processes (52). Hence, disruptions in these pathways emanating from left temporal degeneration may be susceptible to functional impairment as observed in MCI. Interestingly, in case of temporal lobe epilepsy with hippocampal atrophy morphometric changes in the cerebellum have been described before (53). Task-independent co-activation with the left and the right temporal seeds was found in lobule IX of the cerebellum, which has been described as being part of the default-mode network (DMN) (54). In the parietal lobe we further found pronounced co-activation with structures of the DMN such as the angular gyrus, which is considered to be a connecting hub and involved in memory functions, theory of mind and social cognition (55, 56). Altered DMN connectivity has been consistently reported in Alzheimer's disease, and based on longitudinal studies the strength of interregional connectivity seems to decrease when MCI patients convert to dementia (11). While targeted studies need to further evaluate the specific diagnostic and prognostic value of network alterations for the course of MCI, the functional connectivity profiles of the temporal seeds offer a framework to characterize possible network damage in MCI and neural correlates of subsequent MCI-related neuropsychological deficits. The delineated convergence patterns of functional connectivity indicate differential networks formed by each atrophy seed and related to specific behavioral symptoms. While the left temporal seed had a stronger convergence with a fronto-temporal network associated with speech generation, on the contralateral site connectivity patterns encompassed rather striatal and orbitofrontal regions indicating a role in emotional control.

### Functional Connectivity of the Parietal Atrophy Seed

The precuneus and PCC as well as the hippocampus are part of the “rich club” of highly interconnected hub centers of the brain (13, 57), which is known for its integrative long-range connections and plays a major role in the brains ability to perform cognitive functions. The PCC and precuneus are vital parts of the DMN and abnormalities have been reported in a range of different diseases such as Alzheimer's disease (11, 58–61), schizophrenia, autism and depression (56, 62). The ventral PCC is associated with internally focused states and the dorsal part with externally directed attention (63). The precuneus is involved in monitoring and execution of spatially guided behavior, mental imagery and episodic memory retrieval (64, 65). The PCC and precuneus share intensive connections with each other and are considered as the “posterior” part of the DMN (66). Unsurprisingly, the parietal seed demonstrated a much more pronounced and widespread task-independent than task-dependent connectivity pattern including the medial prefrontal cortex, fronto-insular cortex, middle and inferior temporal gyri, hippocampus, amygdala and the posterior part of the cerebellum. The difference between those two modes illustrates the behavioral specificity of this cluster and emphasizes its role in introspection and non-directional cerebral activity. Especially functional convergence of the PCC and the ventromedial prefrontal cortex is suggestive of DMN activity (67). Task-dependent co-activation was located in the middle orbital and superior medial gyrus, left middle frontal gyrus, inferior parietal lobe, hippocampus and amygdala. Behavioral decoding showed for the parietal seed compared to the temporal clusters a significant probability for the domains of social cognition and motion perception, and the paradigms of theory of mind, semantic discrimination, episodic recall and passive listening. This reflects the parietal cluster's role in the DMN as well as in memory functions and understanding of speech. The overlap between the domain of social cognition and areas active during resting state has been described before and is being interpreted that both social cognitive processes and the resting state are linked to introspection (68). Given the DMN's known function in autobiographical remembering (18) memory deficits in MCI may also be linked to parietal volume reductions and ensuing functional network disruptions. In summary, the significance of morphological changes in the precuneus and the PCC particularly lies in their role as integrative hub regions in the brain. Network dysfunction in the case of damage to hub regions is more detrimental than in the case of less interconnected structures (57). Given the broad overlap of structures of the DMN and epicenters of connectivity, disruptions of these hubs will likely compromise network communication and the brain's ability to integrate information, which seems to be in particular at risk in case of MCI.

### Effects of Aging on Functional Connectivity

Next, we investigated age-related connectivity of MCI-typical atrophy regions and found decreasing co-activation between temporal seeds and the medial frontal, medial parietal and middle and inferior temporal regions with higher age, while connectivity to lateral prefrontal and parietal cortex increased in older subjects. The shift of higher functional connectivity in older individuals from the OFC to the dorsolateral prefrontal cortex (DLPFC) is in line with previous literature that described greater age differences in OFC-sensitive cognitive tasks in comparison to DLPFC tasks supporting the notion that the OFC is susceptible to earliest age-related changes (69). Furthermore, a posterior/anterior-shift of task-dependent activation has been described with aging in which activation shifts from parietal and occipital regions toward prefrontal areas implicating compensatory recruitment of prefrontal regions due to age-related sensory-processing deficits (70, 71). Age-related increases in brain activity, however, do not only involve the frontal lobe, but have also been reported for parietal structures (70, 71). Our analysis showed a pattern of decreasing task-independent connectivity between the temporal seeds and parietal, medial orbitofrontal and temporal regions with higher age, whereas connectivity to the lateral PFC and inferior parietal regions (supramarginal gyrus) increased. Hence, the age-dependent posterior-anterior shift may to some extent also apply to connectivity changes at rest, as also previously reported by Roski et al. (72). Interestingly, temporal seeds further demonstrated an increase of connectivity with cerebellar lobules VI and VII suggesting that the cerebellum might be involved in adaptive processes of the aging brain. However, it is important to note that we cannot infer based on our current analysis in healthy individuals if such a compensation strategy is also maintained in MCI or already overcome by MCI-related pathological changes. On the other hand, given that patients with MCI exhibit cognitive deficits without relevant impact on daily living, it seems reasonable that such a neural reorganization pattern may contribute to preserve functional maintenance in MCI, which needs to be addressed in future imaging studies comparing MCI and healthy aging.

Finally, we found mainly positive correlations between age and connectivity of the parietal cluster with the middle occipital gyrus, middle temporal gyrus, and angular gyrus. Decreasing connectivity with higher age could only be found in the insula, in contrast to the more widespread pattern of age-related decline of connectivity of the temporal clusters. In accordance with this, the PCC is known for its relatively good preservation in age (73), and seems to be subject to relatively fewer alterations in normal aging than pathological processes such as Alzheimer's disease and MCI (74). Additionally, the increasing connectivity within parietooccipital areas with higher age may also reflect an age-dependent loss of functional specialization (e.g., sensory-processing functions) in terms of a dedifferentiation process and decrease in intra-network distinctiveness. This notion of neural dedifferentiation has been postulated for cognitive functioning in the aging brain [e.g., (75)] and was also shown for resting-state connectivity (72), which may likely be augmented due to MCI-related morphological changes and network disruptions constricting the brains ability to adapt to the aging process.

## Limitations and Conclusions

Based on structurally-affected areas in MCI that are consistent across studies, we investigated ensuing functional alterations emanating from this degenerative pattern as well as their possible clinical relevance. However, it is important to note that our approach relies upon networks derived from healthy brain functioning and likely interfering with structural damage typically observed in MCI. Hence, it does not allow conclusions regarding the degree of disruptions in functional networks, disease-stage dependent effects, or any causality, but rather delineates circuits formed by nodes affected in MCI and potentially disrupted in this disease. The knowledge of these connections and their clinical impact is important and relevant as they give further insights into the functional architecture of cognitive impairment. This also pertains to our correlation analysis between functional connectivity and age in healthy individuals as discussed above. While we were able to outline age-dependent task-free functional alterations in an aging population derived from the 1000BRAINS study, this was not possible for the meta-analytical task-dependent analysis, which would have allowed further insights on age-related co-activation patterns under cognitive demand. In addition, it is noteworthy that the large database of the 1000BRAINS study was designed to allow investigations into age-related variability in brain structure and function in the general population with a focus on the aging brain. Nevertheless, we cannot rule out a certain sampling bias in particular pertaining to the here used cohort of older healthy subjects that may affect generalizability to the general aging population. Another limitation is the fact that MCI is a heterogenous syndrome with different etiologies possibly underlying mental decline, whereas precise diagnostic evaluation encompasses the measurement of biomarkers like amyloid and tau-proteins in the cerebrospinal fluid. On the other hand, given that the here used definition of MCI was not bound to a certain etiology and functional analyses were based on shared morphologically affected regions, our findings relate to network patterns associated with these nodes as a common denominator of cognitive decline (8). Hence, the here delineated functional profiles in task-free and task-driven states offer a framework to characterize the pathophysiological impact of atrophy loci found in patients with cognitive impairment. Functional connectivity analysis in concordance with behavioral characterization and previous literature demonstrated susceptibility of functional disruption in networks responsible for language generation, emotional control, theory of mind as well as non-directional cerebral activity. These areas may also have an increased vulnerability to disease spreading along network lines in neurodegenerative diseases compromising integrative capabilities of the brain, which are essential for global cognitive functioning, as well as age-related adaptive processes. These findings help to understand the ensuing clinical relevance of structural damage in MCI beyond memory deficits.

## Data Availability Statement

Datasets supporting the conclusions of this article will be made available to qualified researchers on request.

## Ethics Statement

The studies involving human participants were reviewed and approved by the University of Duisburg-Essen. The patients/participants provided their written informed consent to participate in this study.

## Author Contributions

SE, AL, PF, KR, and ID conceived and designed the study. GS, FH, SE, and ID analyzed the data. GS, KR, and ID interpreted the data. GS drafted the manuscript. SE, SC, TN-J, AL, JS, KR, and ID critically reviewed the manuscript.

### Conflict of Interest

The authors declare that the research was conducted in the absence of any commercial or financial relationships that could be construed as a potential conflict of interest. The reviewer KR declared a past co-authorship with several of the authors AL, PF, and SE to the handling Editor.
