# Supplementary material for: Functional Characterization of Atrophy Patterns Related to Cognitive Impairment
Source: Front Neurol. 2020 Jan 24;11:18. doi: 10.3389/fneur.2020.00018 (PMC6993791; doi:10.3389/fneur.2020.00018)
Supplement: Supplementary file 1 [file Data_Sheet_1.PDF]

Supplementary Material

Table S-1: Task-dependent MACM functional connectivity of MCI-atrophy seeds

Cluster #	k_E	MNI co-ordinates*			Lat.	Macroanatomical and cytoarchitectonic region
		x	y	z		
Temporal left						
Cluster 1	17851	-22	-6	-18	L	Amygdala (LB, CM, SF), thalamus, caudate nucleus, putamen, hippocampus (CA, DG, SUB, HATA), parahippocampal gyrus<br>Fusiform gyrus, inferior, middle and superior temporal gyrus, inferior occipital gyrus, cerebellum (VI),<br>Precentral gyrus, inferior frontal gyrus (Area 44, 45), insula, parietal operculum (SII)
		24	-4	-18	R	Amygdala (LB, SF, CM), thalamus, putamen, hippocampus (CA, DG, SUB, HATA), middle and superior frontal gyrus, Inferior frontal gyrus, insula
Cluster 2	1473	42	-52	-22	R	Fusiform gyrus, middle and inferior temporal gyrus, inferior occipital gyrus, cerebellum (VI)
Cluster 3	1441	-2	12	54	L/R	Posterior medial frontal gyrus (SMA)
Cluster 4	373	0	46	-16	L/R	Frontal pole, rectal gyrus, mid-orbital gyrus
Cluster 5	344	-6	-52	20	L	Precuneus, PCC
Cluster 6	258	-48	-68	22	L	Angular gyrus, IPL
Temporal right						
Cluster 1	14119	24	-4	-18	R	Amygdala (LB, SF, CM), thalamus, caudate nucleus, putamen, pallidum, hippocampus (CA, SUB, EC, HATA), inferior frontal gyrus, insula, cerebellum (lobule VI)
		-22	-6	-18	L	Amygdala (LB, SF, CM), caudate nucleus, putamen, pallidum, hippocampus (CA, SUB, HATA), thalamus<br>Fusiform gyrus, parahippocampal gyrus<br>inferior frontal gyrus (Area 44, 45), insula
Cluster 2	1309	-2	18	46	L/R	Posterior medial frontal gyrus (SMA)
Cluster 3	881	42	-50	-20	R	Fusiform gyrus, inferior occipital, inferior temporal gyrus
Cluster 4	803	4	52	-6	L/R	Frontal pole, rectal gyrus, mid-orbital gyrus
Cluster 5	614	46	10	28	R	Inferior frontal gyrus (p. opercularis, p.triangularis)
Precuneus						
Cluster 1	3120	0	-54	32	L/R	Precuneus, MCC, PCC, calcarine gyrus
Cluster 2	2243	-2	50	-8	L/R	Frontal pole, middle orbital cortex, rectal gyrus; superior medial gyrus, ACC
Cluster 3	1271	-52	-62	30	L	Angular gyrus, IPL
Cluster 4	826	54	-56	28	R	Angular gyrus, IPL
Cluster 5	589	-24	24	48	L	Middle frontal gyrus, superior frontal gyrus
Cluster 6	168	58	-6	-22	R	Middle temporal gyrus
Cluster 7	165	-22	-10	-14	L	Amygdala (CM), hippocampus (CA)
Cluster 8	154	24	-8	-12	R	Amygdala (CM), hippocampus (CA)

Task-based (MACM) functional connectivity maps of each MCI-atrophy seed (cluster-level FWE corrected at $p<0.05$; cluster-forming threshold $p<0.001$). *Cluster-maxima in MNI space. k_E: cluster extent; Lat.: laterality; L: left; R: right; CA: cornu ammunis; EC: entorhinaler cortex; SUB: subiculum; DG: dentate gyrus; HATA: hippocampus–amygdala-transition-area; LB: laterobasal; SF: superficial; CM: centromedial, ACC: anterior cingulate cortex, MCC: middle cingulate cortex; PCC: posterior cingulate cortex, SPL: superior parietal lobule; IPL: inferior parietal lobule.

Table S-2: Comparison of MACM functional connectivity maps of MCI-atrophy seeds

Cluster #	k_E	MNI co-ordinates*			Lat.	Macroanatomical and cytoarchitectonic region
		x	y	z		
Contrast: Temporal right < temporal left						
Cluster 1	6018	-22	-10	-30	L	Hippocampus (CA, SUB, FD), amygdala (LB, SF, CM); thalamus, insula, inferior, middle and superior temporal gyrus, fusiform gyrus, cerebellum (VI)
Cluster 2	766	-50	20	10	L	Inferior frontal gyrus (p. triangularis, orbitalis)
Cluster 3	362	40	-60	-24	R	Cerebellum (VI, VIIa), fusiform gyrus
Cluster 4	283	-46	-68	-18	L	Fusiform gyrus, inferior occipital gyrus, middle temporal gyrus
Cluster 5	246	54	6	-24	R	Middle and superior temporal gyrus
Cluster 6	224	-50	-68	32	L	Angular gyrus, IPL
Cluster 7	84	0	-8	56	L	Posterior-medial frontal
Contrast: Temporal right > temporal left						
Cluster 1	4565	24	-12	-30	R	Hippocampus (SUB, EC, HATA, CA), amygdala (LB, SF), parahippocampal gyrus, caudate nucleus, putamen, pallidum, olfactory cortex, insula, inferior frontal gyrus (p. orbitalis), thalamus
Cluster 2	294	6	50	-4	R	Middle orbital gyrus
					L	Middle orbital gyrus
Cluster 3	283	-10	8	-12	L	Caudate nucleus, putamen, pallidum
Cluster 4	106	4	8	38	R	Middle cingulate cortex
Contrast: Temporal right and left > precuneus						
Cluster 1	4332	-24	-14	-25	L	Hippocampus (CA, SUB, HATA, DG), amygdala (CM, LB, SF), parahippocampal gyrus, fusiform gyrus, putamen, inferior occipital gyrus cerebellum (VI, VIIa)
Cluster 2	3529	26	-10	-25	R	Hippocampus (CA, SUB, HATA, DG), amygdala (CM, LB, SF), parahippocampal gyrus, caudate nucleus, pallidum
Cluster 3	779	40	-54	-15	R	Fusiform gyrus, inferior temporal gyrus, inferior occipital gyrus, cerebellum (Lobule VI)
Cluster 4	325	2	-2	71	R	MCC, posterior medial gyrus
					L	MCC, superior medial gyrus
Cluster 5	197	-28	22	-3	L	Insula lobe, inferior frontal gyrus (p. orbitalis)
Cluster 6	124	-46	28	15	L	Inferior frontal gyrus (p. triangularis)
Cluster 7	107	30	12	9	R	Putamen, insula
Contrast: Temporal right and left < precuneus						
Cluster 1	2885	2	-58	22	R	Precuneus,PCC, MCC
					L	Precuneus, PCC, MCC, calcarine gyrus
Cluster 2	1368	2	54	8	L	Middle orbital gyrus, superior medial gyrus, ACC, olfactory cortex
					R	Middle orbital gyrus, superior medial gyrus, ACC
Cluster 3	1123	-52	-62	28	L	Angular gyrus, IPL
Cluster 4	768	54	-56	28	R	Angular gyrus, IPL
Cluster 5	549	-38	20	44	L	Middle and superior frontal gyrus

Contrast and conjunction analysis of task-based (MACM) functional connectivity maps of each MCI-atrophy seed. *Cluster-maxima in MNI space. k_E: cluster extent; Lat.: laterality; L: left; R: right; CA: cornu ammunis; EC: entorhinaler cortex; SUB: subiculum; DG: dentate gyrus; HATA: hippocampus–amygdala-transition-area; LB: laterobasal; SF: superficial; CM: centromedial, ACC; anterior cingulate cortex; MCC: middle cingulate cortex; PCC: posterior cingulate cortex SPL: superior parietal lobule; IPL: inferior parietal lobule.

Table S-3: Task-independent resting-state functional connectivity of MCI-atrophy seeds

Cluster #	k_E	MNI co-ordinates* x	y	z	Lat.	Macroanatomical and cytoarchitectonic region
Temporal right						
Cluster 1	26965	26	-14	-22	R	Hippocampus (CA , EC, SUB, DG), amygdala (LB, SF, CM), rectal gyrus, middle temporal gyrus, fusiform gyrus, pallidum, putamen, precuneus, thalamus, PCC, lingual gyrus, parahippocampal gyrus, inferior frontal gyrus (p. orbitalis)
					L	Hippocampus (CA, EC, SUB, DG), amygdala (LB, SF, CM), fusiform gyrus, rectal gyrus, thalamus, precuneus, PCC, lingual gyrus, parahippocampal gyrus
Cluster 2	1721	50	-62	22	R	Angular gyrus, IPL, middle temporal, superior occipital gyrus
Cluster 3	1521	-46	-76	34	L	Angular gyrus, IPL
Cluster 4	663	20	30	40	R	Superior frontal gyrus
Cluster 5	630	-8	-52	-44	L, R	Cerebellum (IX, X)
Cluster 6	314	-22	24	38	L	Superior frontal gyrus
Cluster 7	302	-64	-6	28	L	Precentral gyrus, postcentral gyrus
Cluster 8	246	64	-6	20	R	Postcentral gyrus
Cluster 9	98	-12	-86	-40	L	Cerebellum (VIIa)
Cluster10	94	-32	32	-16	L	Inferior frontal gyrus (p. orbitalis)
Cluster11	87	-40	-32	68	L	Postcentral gyrus
Cluster12	67	36	-8	16	R	Insula lobe
Cluster13	60	-6	-26	56	L	Paracentral lobule
Cluster14	46	14	-84	-42	R	Cerebellum (VIIa)
Temporal left						
Cluster 1	21761	-44	4	-20	L	Hippocampus (CA, SUB, DG, EC), amygdala (LB, SF, CM), rectal gyrus, fusiform gyrus, thalamus, cerebellum (IV, V), precuneus, PCC, parahippocampal gyrus, insula lobe, inferior, middle and superior temporal gyrus
					R	Hippocampus (CA , EC, SUB, DG), amygdala (LB, SF, CM), thalamus, precuneus, PCC, cerebellum (V, VI), insula lobe, middle and superior temporal gyrus, fusiform gyrus
Cluster 2	1841	2	38	-22	R	Rectal gyrus, olfactory cortex
Cluster 3	1170	-46	-62	24	L	Angular gyrus, IPL, middle temporal gyrus
Cluster 4	289	8	-52	-42	R, L	Cerebellum (IX)
Cluster 5	243	58	-66	22	L	Angular gyrus, middle temporal gyrus
Cluster 6	175	32	-80	-36	R	Cerebellum (VIIa)
Cluster 7	55	1	-39	-20	R	Cerebellum (vermis)
Cluster 8	50	-30	-82	-34	L	Cerebellum (VIIa)
Precuneus						
Cluster 1	23967	-2	-58	34	L	Precuneus, middle orbital gyrus, rectal gyrus, MCC, PCC, middle and superior frontal gyrus, superior medial gyrus, middle frontal gyrus, hippocampus (SUB, CA, EC), amygdala (SF, LB), lingual gyrus, ACC, calcarine gyrus
					R	Precuneus, middle orbital gyrus, MCC, PCC, rectal gyrus, middle and superior frontal gyrus, hippocampus (SUB, CA, EC), amygdala (SF, LB), lingual gyrus, ACC, calcarine gyrus
Cluster 2	3348	-62	-14	-20	L	Middle temporal gyrus, inferior temporal gyrus
Cluster 3	3279	-46	-68	34	L	Angular gyrus, IPL, superior parietal, middle occipital gyrus
Cluster 4	2998	62	-8	-20	R	Middle temporal gyrus, inferior temporal gyrus
Cluster 5	2749	56	-62	26	R	Angular gyrus, IPL
Cluster 6	1655	16	-86	-40	R	Cerebellum (VIIa)
Cluster 7	1375	-14	-86	-40	L	Cerebellum (VIIa)
Cluster 8	890	8	-52	-44	R, L	Cerebellum (IX)
Cluster 9	378	28	18	-20	R	Inferior frontal gyrus (p. orbitalis), insula lobe
Cluster10	273	-26	16	-22	L	Inferior frontal gyrus (p. orbitalis), insula lobe

Task-free (resting-state) functional connectivity maps of each MCI-atrophy seed (FWE corrected $p<0.05$ at voxel-level). *Cluster-maxima in MNI space. k_E: cluster extent; Lat.: laterality; L: left; R: right; CA: cornu ammunis; EC: entorhinaler cortex; SUB: subiculum; DG: dentate gyrus; HATA: hippocampus–amygdala-transition-area; LB: laterobasal; SF: superficial; CM: centromedial, MCC: middle cingulate cortex; SPL: superior parietal lobule; IPL: inferior parietal lobule.

Table S-4: Comparison of resting-state functional connectivity maps of MCI-atrophy seeds

Cluster #	k_E	MNI co-ordinates*			Lat.	Macroanatomical and cytoarchitectonic region
		x	y	z		
Contrast: Temporal left > temporal right						
Cluster 1	1886	-56	-20	-8	L	Middle temporal gyrus, angular gyrus, IPL
Cluster 2	647	-38	20	-18	L	Inferior frontal gyrus (p. orbitalis, p. triangularis), insula, putamen
Cluster 3	615	-10	58	26	L	Superior medial gyrus, superior frontal gyrus
Cluster 4	407	52	-20	-12	R	Middle temporal gyrus
Cluster 5	311	-44	2	-38	L	Inferior temporal gyrus
Cluster 6	295	30	-80	-36	R	Cerebellum (VIIa)
Contrast: Temporal right > temporal left						
Cluster 1	1008	46	-74	28	R	Angular gyrus, IPL, middle temporal gyrus
Cluster 2	663	6	-34	34	R	MCC, lingual gyrus, cuneus, precuneus
Cluster 3	607	26	32	-16	R	Middle orbital gyrus, olfactory cortex, caudate nucleus
					L	Caudate nucleus, putamen
Cluster 4	486	24	24	38	R	Middle frontal gyrus, superior frontal gyrus
Cluster 5	443	24	-28	-6	R	Thalamus, hippocampus, putamen
Cluster 6	399	-36	-82	26	L	Middle occipital gyrus
Cluster 7	297	18	-42	-46	R	Cerebellum (IX, X)
Cluster 8	210	48	-46	-18	R	Fusiform gyrus, inferior temporal gyrus
Contrast: Temporal left and right > precuneus						
Cluster 1	8053	-24	-8	-22	L	Hippocampus (EC, SUB, CA, FD), amygdala (LB, SF, CM), fusiform gyrus, superior and inferior temporal gyrus
					R	Hippocampus (CA, SUB, EC, FD), amygdala (LB, SF, CM). fusiform gyrus, superior temporal gyrus
Cluster 2	425	-48	-12	28	L	Postcental gyrus, Rolandic Operculum, insula lobe
Cluster 3	211	64	-6	32	R	Postcentral gyrus
Contrast: Temporal left and right < precuneus						
Cluster 1	2669	-22	62	8	L	Superior and middle frontal gyrus, superior orbital gyrus, ACC
Cluster 2	1835	-8	-66	36	L	Precuneus, PCC, MCC, cuneus
					R	Precuneus, MCC, PCC, cuneus
Cluster 3	1675	18	30	52	R	Superior frontal gyrus, superior medial gyrus, ACC
Cluster 4	1160	48	-68	-4	R	Cerebellum (VIIa Crus I u. II)
Cluster 5	943	-46	-66	42	L	Angular gyrus, IPL
Cluster 6	928	-24	-84	-30	L	Cerebellum (VIIa Crus I u. II)
Cluster 7	894	54	-56	32	R	Angular gyrus, IPL, supramarginal gyrus
Cluster 8	825	38	14	46	R	Middle frontal gyrus
Cluster 9	257	-64	-36	-16	L	Middle temporal gyrus, inferior temporal gyrus
Cluster10	254	66	-34	-8	R	Middle temporal gyrus
Cluster11	114	12	-102	2	R	Lingual gyrus
Cluster12	109	6	-6	4	R	Thalamus
Cluster13	103	-4	-12	8	L	Thalamus

Contrast and conjunction analysis of task-free (resting-state) functional connectivity maps of each MCI-atrophy seed. *Cluster-maxima in MNI space. k_E: cluster extent; Lat.: laterality; L: left; R: right; CA: cornu ammunis; EC: entorhinaler cortex; SUB: subiculum; DG: dentate gyrus; HATA: hippocampus–amygdala-transition-area; LB: laterobasal; SF: superficial; CM: centromedial, ACC: anterior cingulate cortex, MCC: middle cingulate cortex; PCC: posterior cingulate cortex; SPL: superior parietal lobule; IPL: inferior parietal lobule.
